# Relationship of the Degree of Sarcopenia with the Severity of Nonalcoholic Fatty Liver Disease and Cardiometabolic Risk in Adolescents

**DOI:** 10.3390/life14111457

**Published:** 2024-11-10

**Authors:** Yoowon Kwon, Jin A Chung, You Jin Choi, Yoo Min Lee, So Yoon Choi, In Hyuk Yoo, Tae Hyeong Kim, Su Jin Jeong

**Affiliations:** 1 Department of Pediatrics, Chungnam National University Sejong Hospital, Chungnam National University School of Medicine, Sejong 30099, Republic of Korea; youyisi68@gmail.com; 2 Department of Pediatrics, CHA Bundang Medical Center, CHA University School of Medicine, Seongnam 13496, Republic of Korea; jinach88@naver.com; 3 Department of Pediatrics, Ilsan Paik Hospital, Inje University College of Medicine, Ilsan 10380, Republic of Korea; jeania83@gmail.com; 4 Department of Pediatrics, Soonchunhyang University Bucheon Hospital, Soonchunhyang University College of Medicine, Bucheon 14584, Republic of Korea; flana@schmc.ac.kr; 5 Department of Pediatrics, Kosin University Gospel Hospital, Kosin University College of Medicine, Busan 49267, Republic of Korea; thdbs1206@hanmail.net; 6 Department of Pediatrics, Seoul St. Mary’s Hospital, College of Medicine, The Catholic University of Korea, Seoul 06591, Republic of Korea; yoohymn@naver.com; 7 Department of Pediatrics, Kyung Hee University Hospital at Gangdong, Kyung Hee University College of Medicine, Seoul 05278, Republic of Korea; pgnkim@khu.ac.kr

**Keywords:** nonalcoholic fatty liver disease, skeletal muscle mass, sarcopenia, triglyceride and glucose index, atherogenic index of plasma

## Abstract

The association between nonalcoholic fatty liver disease (NAFLD) and sarcopenia has been suggested. We investigated sarcopenia’s impact on NAFLD severity and its relationship with cardiometabolic risk in adolescents. We conducted a retrospective study on 122 patients aged 13–18 years and diagnosed with both NAFLD and sarcopenia by laboratory tests, abdominal ultrasound (US), and multifrequency bioelectrical impedance analysis. Sarcopenia was stratified into tertiles based on the skeletal muscle-to-fat ratio (MFR), NAFLD severity was established by the US, and cardiometabolic risk was assessed by the triglyceride–glucose (TyG) index and the atherogenic index of plasma (AIP). Compared with the other patients, those in the lower MFR tertiles exhibited a greater severity of NAFLD (*p* < 0.001) and significantly higher TyG index and AIP. The independent effect of MFR was observed to have a negative correlation with the severity of NAFLD (*p* < 0.001). Based on the aforementioned results, the degree of sarcopenia can be considered as one of the risk factors of severe NAFLD and might be an indicator of cardiometabolic risk in adolescents. Weight training to reach the amount of muscle mass could be included in the treatment strategies to improve or prevent NAFLD in adolescents with sarcopenia.

## 1. Introduction

Nonalcoholic fatty liver disease (NAFLD) has emerged as a significant health concern worldwide, particularly among children and adolescents, along with the rise in obesity and metabolic syndrome (MetS) [1,2,3,4]. The well-known risk factors that play a significant role have been central obesity and insulin resistance [5,6,7], but recent attention has been paid to sarcopenia as an additional risk factor for NAFLD [8,9]. Sarcopenia, which is characterized by the loss of skeletal muscle mass (SMM), strength, and function, is a fragile condition that is associated with an increased risk of poor health outcomes. It was traditionally considered to affect only the elderly secondary to age-related changes in body composition, including decreased muscle mass and diminished resting metabolic rate. Studies in adults have linked sarcopenia with diabetes mellitus, MetS, and cardiovascular disease (CVD) [10,11,12]. However, sarcopenia has been recently observed in relatively young individuals, especially as the obesity rates increase [13,14]. Sarcopenic obesity (SO) is defined as the coexistence with increased fat mass [15].

In a previous study, we demonstrated that reduced SMM was an independent risk factor of pediatric NAFLD [16]. Building upon this, our aim in this present study was to further evaluate the impact of sarcopenia degree on the severity of NAFLD in adolescents. In addition, we sought to elucidate the relationship between sarcopenia degree and cardiometabolic risk in this population. To achieve these, we used the triglyceride–glucose (TyG) index and the atherogenic index of plasma (AIP), which are recognized optimal indicators of insulin resistance and CVD [17,18,19,20,21]. By incorporating these indices into our analysis, we aimed to gain a more comprehensive understanding of the association among sarcopenia, NAFLD severity, and cardiometabolic risk factors in adolescents.

## 2. Materials and Methods

### 2.1. Subjects

A retrospective study was conducted on patients who visited the pediatric gastrointestinal clinic at two general hospitals (CHA Bundang Medical Center and Chungnam National University Sejong Hospital) from February 2020 to December 2023 because of abnormal liver function tests. Adolescents aged 13–18 years who were finally diagnosed with NAFLD; had comprehensive data, including laboratory tests, abdominal ultrasound (US), and multifrequency bioelectrical impedance analysis (BIA) (InBody720 or Inbody570; Biospace, Seoul, Republic of Korea); and were defined as having sarcopenia were included for analysis. Patients with other confirmed diseases that cause liver function test elevation (e.g., hepatotropic virus infections, systemic infections, autoimmune hepatitis, Wilson disease, alcohol consumption, and other toxic hepatitis) and chronic diseases, such as immunosuppression or malignancy, were excluded. Patients with comorbid pathological obesity secondary to endocrine disorders, genetic diseases, and central nervous system disorders were also excluded.

This study was approved by the institutional review boards of CHA University (No. 2023-03-001) and Chungnam University (No. 2023-07-002). Written informed consent documents were obtained from the parents or guardians of all participating adolescents.

### 2.2. Anthropometric Measurements

To assess body composition, we performed a BIA test on each patient, who was asked to maintain a stance with legs apart on the machine for approximately 2 min, with arms slightly separated from the trunk, and while wearing light indoor clothes and no shoes. The patients were instructed to hold the handles of the analyzer to achieve contact of each limb with the electrodes. Various parameters, including height (cm), weight (kg), body mass index (BMI in kg/m^2^), body fat mass (kg), segmental lean muscle mass (kg), and percentage of body fat and lean muscle mass (%), were automatically measured.

### 2.3. Definition of Skeletal Muscle Mass Values and Sarcopenia

The BIA technique has been employed in recent studies to estimate appendicular SMM (ASM, kg) because it has a strong correlation with dual-energy X-ray absorptiometry and has been validated for the assessment of body composition [16,22]. Using the BIA results, we calculated ASM as the sum of the SMM of the four limbs under the assumption that all nonfat and nonbone tissues were skeletal muscles [16,23].

Skeletal muscle-to-fat ratio (MFR) was calculated, according to McCarthy et al. [23], by dividing ASM by body fat mass. Based on previous studies, the MFR cutoff value for sarcopenia in this study was defined as 1.155 for boys and 0.723 for girls and was calculated as follows: mean value −1 SD of the MFR for the third BMI quintile of Korean children and adolescents aged 10–18 years [16,23].

### 2.4. Clinical and Laboratory Assessments

Each subject completed a past medical history questionnaire and underwent anthropometric assessment and laboratory tests. Venous samples were obtained after 8 h of fasting. The laboratory tests included serum alanine aminotransferase (ALT), aspartate aminotransferase, and gamma-glutamyltransferase; total cholesterol, triglyceride (TG), high-density lipoprotein cholesterol (HDL-C), and low-density lipoprotein cholesterol (LDL-C); fasting glucose; hepatitis B surface antigen and antibodies to hepatitis A, B, and C virus; ceruloplasmin; and antinuclear antibody. Blood samples were collected in separator tubes containing silica and a gel clot (Becton, Dickinson and Company, Franklin Lakes, NJ, USA), centrifuged, and analyzed within 2 h. All laboratory tests were performed using standard methods.

### 2.5. Sonographic Evaluation of Nonalcoholic Fatty Liver Disease and Fatty Liver Severity

Experienced pediatric radiologists, who were blinded to the clinical and laboratory results of the patients, performed US to diagnose fatty liver. The sonographic diagnosis was established based on characteristics such as bright liver, which indicated diffusely increased liver parenchymal echogenicity, in comparison with the adjacent kidney and spleen, without focal lesions, increased attenuation of the US beam, or diminished sonographic visualization of the portal and hepatic veins [24,25,26]. In accordance with the criteria published by Saadeh et al., NAFLD severity was semiquantitatively graded as mild (grade 1), moderate (grade 2), or severe (grade 3) [8,27].

### 2.6. Calculation and Reference for the Triglyceride–Glucose Index and Atherogenic Index of Plasma

The TyG index was calculated as follows: ln [(fasting TG (mg/dL) × fasting glucose (mg/dL)/2] [28,29]. The reference values for the TyG index were derived from a population-based study on Korean children and adolescents aged 10–18 years by Yoon et al., who reported a median TyG index of 8.07 for boys and 8.14 for girls [20]. The AIP was calculated as follows: log (TG/HDL-C) [30,31]. Based on previous studies, CVD risk was assessed based on the AIP range, as follows: low risk (−0.3–0.1), medium risk (0.1–0.24), or high risk (>0.24) [17,18,19].

### 2.7. Grouping of the Study Population, According to Sarcopenia Status

Patients were stratified into tertiles according to sarcopenia status on MFR for each sex. The group with the lowest MFR was defined as tertile 1 or severe sarcopenia group, whereas the group with the highest MFR was defined as tertile 3 or mild sarcopenia group.

### 2.8. Statistical Analysis

The data were analyzed using descriptive statistics and were presented as mean and standard deviation or proportion. Intergroup comparisons of the mean values of the continuous variables were conducted by analysis of variance. The chi-square test was employed to compare categorical variables, which were expressed as percentages. The relationship between US-graded NAFLD severity and clinical variables was examined using Kendall’s and Spearman’s rank correlation analyses. The association between NAFLD severity and MFR was assessed by ordinary regression analysis, which included calculations of β coefficients, standard errors, and 95% confidence interval. *p* values ≤ 0.05 were considered statistically significant. All statistical analyses were performed using IBM SPSS Statistics 25.0 (IBM^®^ SPSS^®^ Statistics Server, Armonk, NY, USA).

## 3. Results

### 3.1. Comparison of Baseline Characteristics of the Study Population, According to Sarcopenia Status

A total of 122 adolescents who were concurrently diagnosed with sarcopenia and NAFLD were enrolled in this study. Table 1 summarizes the baseline demographic and clinical characteristics of the three groups. The three groups showed no marked differences in sex distribution and mean age; the mean MFR was 0.56 for tertile 1 (0.59 for men, 0.46 for women) and 0.88 for tertile 3 (0.94 for men, 0.66 for women). Compared with the other groups, the tertile 1 group had significantly higher ALT levels and lower HDL-C levels. Serum TG, LDL-C, and glucose levels did not show a clear trend; however, the tertile 3 group had a significantly lower serum TG level. 

### 3.2. Association Between Nonalcoholic Fatty Liver Disease Severity and Sarcopenia Status

**Figure 1:**
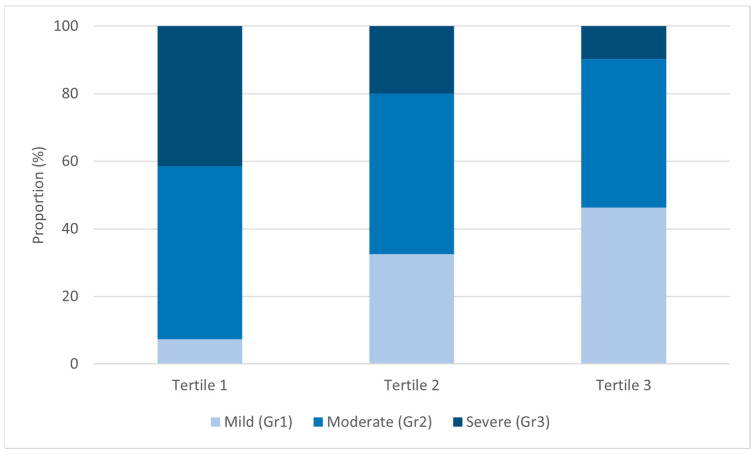
US-graded severity of NAFLD according to MFR tertiles.

The severity of NAFLD was significantly higher in the patients in the lower MFR tertiles (*p* < 0.001), with the proportion of severe NAFLD being 41.5% in tertile 1, 20% in tertile 2, and 9.8% in tertile 3 (Figure 1). In the correlation analysis, NAFLD severity was significantly correlated with BMI (Kendall’s Tau b = 0.141, *p* = 0.046, Spearman’s rho = 0.186, *p* = 0.040) and MFR (Kendall’s Tau b = −0.283, *p* < 0.001, Spearman’s rho = −0.363, *p* < 0.001) (Table 2).

To verify the independent effect of sarcopenia on NAFLD severity, ordinal logistic regression analysis was performed. The results (Table 3) showed a significant negative correlation between the severity of NAFLD and MFR, while BMI is not significant; for each unit increase in the MFR, the probability of having severe NAFLD significantly decreased by 4.154 times (*p* = 0.001).

### 3.3. Correlation of Cardiometabolic Risk with Sarcopenia Status

**Figure 2:**
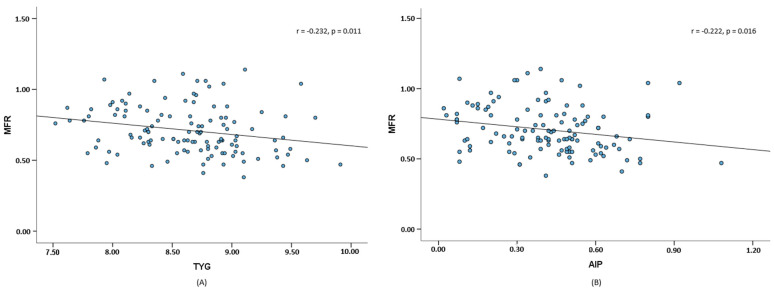
Scatter plot and fitted lines of MFR, TyG (**A**), and AIP (**B**).

Compared with the other groups, the tertile 1 group had a significantly higher TyG index and AIP (Table 4). Compared with Korean adolescents [20], all patients in this study had higher median TyG index and AIP values associated with high CVD risk [17,18,19]. Moreover, as shown in Figure 2, the MFR had significant negative correlations with the TyG index (r = −0.232, *p* = 0.011) and AIP (r = −0.222, *p* = 0.016).

## 4. Discussion

Our study revealed significant insights into the relationship among sarcopenia, NAFLD severity, and cardiometabolic risk factors in adolescents. First, we found a significant association between sarcopenia status and NAFLD severity on the US. Compared with adolescents with higher MFR, those with lower MFR (i.e., severe sarcopenia) demonstrated a greater severity of NAFLD. This inverse relationship persisted even after accounting for potential confounding factors, such as BMI, highlighting the independent impact of sarcopenia status on NAFLD severity. Similarly, previous research on Korean adults indicated that compared with individuals without sarcopenia, those with sarcopenia were more likely to have severe NAFLD and that lower quartiles of ASM percentage were linked with greater NAFLD severity [8]. Another Korean nationwide survey indicated that compared with nonsarcopenic individuals, those with lower skeletal muscle index were more prone to advanced fibrosis [13].

Second, we explored the relationship between sarcopenia status and cardiometabolic risk using the TyG index and AIP. The TyG index is recognized as a reliable indicator of insulin resistance and has significant associations with type 2 diabetes mellitus, NAFLD, and MetS in adults [20]. Although research in children and adolescents had been limited, a population-based study on individuals aged 10–18 years in Korea in 2021 highlighted the clinical significance of the TyG index, which showed a stable distribution, regardless of age, sex, and BMI, with median values of 8.07 for boys and 8.14 for girls [20]. Another Korean study [32] suggested that among adolescents aged 10–18 years who had MetS, according to three sets of criteria by Cook et al. [33], de Ferranti et al. [34], and the International Diabetes Federation [35], the cutoff TyG indices were 8.48 (men 8.48, women 8.48); 8.41 (men 8.40, women 8.38); and 8.66 (men 8.66, women 8.61), respectively. In our study, the mean TyG index was high among patients with severe sarcopenia, surpassing the cutoff value for MetS in tertiles 1 and 2. This result was similar to the findings in a previous study, which showed that the TyG index was independently and negatively associated with a low skeletal muscle index among Korean adults [36]. Similarly, AIP has been used as an optimal indicator of the risks of atherosclerosis and CVD [17]. In this present study, all patients had an AIP of >0.24, which indicated high risk of atherosclerosis and CVD, with a notable increase in tertile 1. To the best of our knowledge, this finding was novel, because there had been no direct investigations on the relationship between AIP and sarcopenia.

Sarcopenia, which is caused by adverse muscle changes during the lifespan of an individual, significantly impedes daily activities, mobility, and overall quality of life [37,38,39,40] and has been linked with respiratory, cardiac, and cognitive issues [37,41,42,43]. It is a relatively novel concept in pediatrics because it has been traditionally associated with aging and the elderly [44]. Moreover, research on sarcopenia in children has been hampered by the lack of uniform definitions and limited studies on its potential impact on clinical outcomes. However, the development of sarcopenia is now recognized to begin earlier in life, especially with the rising incidence of SO in children. Obesity worsens sarcopenia by promoting fat infiltration into muscle and reducing physical function; in fact, SO is defined as reduced lean body mass in the context of excess adiposity [37,45,46]. During the COVID-19 pandemic, the rate of sarcopenia and SO in children and adolescents naturally increased because of limited activity levels.

Although the precise underlying mechanism of their close association remains incompletely explored, sarcopenia and NAFLD share several risk factors, including insulin resistance; chronic inflammation; myokines, such as myostatin; dysregulation of adiponectin; physical deconditioning; and deficiencies of nutrients, such as vitamin D [16,47,48]. Given that both the liver and skeletal muscle are target organs for insulin, insulin resistance is believed to be the primary contributing factor [48]. Decreased SMM can induce insulin resistance, thereby promoting adipokine-induced liver damage through heightened inflammation, oxidative stress, mitochondrial dysfunction, and increased ectopic fat accumulation secondary to increased free fatty acids and decreased lipid β oxidation [16,48]. Excessive secretion of cytokines, such as the commonly implicated inflammatory markers interleukin 6 and tumor necrosis factor α [16,48,49,50], further exacerbates chronic inflammation and oxidative stress, resulting in worsened dysregulation of the muscle–liver axis and driving both muscle mass loss and NAFLD progression. Myostatin, which is a critical myokine for regulating muscle mass, was reported to promote protein breakdown, inhibit skeletal muscle growth, and correlate with obesity and insulin resistance [9]. Deletion of myostatin in mice was found to increase muscle mass, reduce adiposity, enhance insulin sensitivity and glucose uptake, and protect against hepatic steatosis [51,52]. Low SMM contributes to physical disability and increases obesity risk, forming a vicious cycle that leads to liver steatosis [16]. Lastly, recent research indicated that vitamin D insufficiency or disrupted signaling pathways might contribute to metabolic disorders that affect both muscle and liver function [9,53]. Further research is necessary to validate and organize these hypotheses on the relationship between low SMM and NAFLD.

In this study, the MFR cutoff value for sarcopenia was based on our previous study [16] and another study on Korean children and adolescents [23]. Muscle mass was measured by BIA, which is known for its good correlation with dual-energy X-ray absorptiometry and offers several advantages of simplicity, speed, and noninvasiveness in pediatric patients [16,22,54].

This study has certain limitations that should be acknowledged. The recruitment of patients meeting the specific criteria for NAFLD, obesity, and sarcopenia was challenging, which limited our sample size. Additionally, we recognize that the cross-sectional design restricts our ability to infer causal relationships. Furthermore, the regional scope of the data, being limited to a specific area in Korea, may affect the generalizability of our findings to other populations. Finally, due to missing data, we could not adjust for all confounding factors, such as insulin levels, which are closely associated with both sarcopenia and NAFLD. Future longitudinal studies with a larger cohort will be necessary to validate these findings and enhance their applicability to a broader population.

In this study that comprehensively evaluated sarcopenia status in terms of its independent impact on NAFLD severity and its relationship with cardiometabolic risk, we aimed to contribute to the growing body of evidence and highlighted the importance of considering skeletal muscle health in the assessment and management of NAFLD in adolescents. Understanding the relationship between sarcopenia and NAFLD severity could have significant clinical implications in terms of the development of novel therapeutic targets and interventions, such as weight training, to mitigate the progression of liver disease in affected adolescents. Exploring the association between sarcopenia and cardiometabolic risk could offer valuable insights into the complex interplay between muscle health and metabolic health in adolescents. This knowledge could ultimately improve targeted strategies for preventing and managing NAFLD and its associated complications, potentially improving the long-term health outcomes and quality of life of this population.

## 5. Conclusions

The degree of sarcopenia can be considered as one of the risk factors of severe NAFLD and might be an indicator of cardiometabolic risk in adolescents. The increasing prevalence of abdominal obesity in this population, which is associated with sarcopenia, is a growing concern, underscoring the crucial role of physical activity education in addressing this issue. Incorporating weight training to enhance muscle mass should be considered as part of treatment strategies aimed at improving or preventing NAFLD and cardiometabolic diseases in adolescents with sarcopenia.

## Figures and Tables

**Table 1:** Baseline characteristics of the study population according to MFR tertiles.

Variables	Tertile 1 (Lowest, *n* = 41)	Tertile 2 (*n* = 40)	Tertile 3 (Highest, *n* = 41)	*p* Value
Demographics				
Age (year)	14.54 ± 1.45	14.95 ± 1.68	15.00 ± 1.34	0.310
Gender, Male (%)	32 (78.0)	31 (77.5)	32 (78.0)	0.998
Anthropometrics				
BMI (kg/m^2^)	30.63 ± 5.41	30.89 ± 3.85	28.27 ± 2.98	0.010
ASM (kg)	20.48 ± 5.86	22.73 ± 4.32	23.79 ± 5.45	0.038
MFR	0.56 ± 0.07	0.70 ± 0.09	0.88 ± 0.15	<0.001
Male	0.59 ± 0.05	0.74 ± 0.05	0.94 ± 0.10	<0.001
Female	0.46 ± 0.04	0.56 ± 0.04	0.66 ± 0.03	<0.001
Biochemistry				
ALT (IU/L)	128.00 ± 134.14	76.00 ± 53.74	70.00 ± 53.32	0.007
TG (mg/dL)	140.08 ± 61.16	145.63 ± 66.87	102.44 ± 6.87	0.002
LDL-C (mg/dL)	109.82 ± 31.15	110.72 ± 25.23	104.53 ± 21.14	0.566
HDL-C (mg/dL)	45.61 ± 6.40	45.80 ± 5.67	46.08 ± 8.29	0.954
Glucose (mg/dL)	99.46 ± 11.79	93.80 ± 11.64	97.51 ± 14.20	0.126

Data are presented as mean ± SD or number (percent). Abbreviation: SD, standard deviation; BMI, body mass index; ASM, appendicular skeletal muscle mass; MFR, skeletal muscle-to-body fat ratio; ALT, alanine aminotransferase; TG, triglyceride; LDL-C, low-density lipoprotein cholesterol; HDL-C, high-density lipoprotein cholesterol.

**Table 2:** Correlation between US-graded severity of NAFLD and clinical variables.

NAFLD Grade	Age	Gender	BMI	MFR	ALT
Kendall’s Tau-b	−0.016	0.059	0.141	−0.283	0.093
*p* value	0.833	0.490	0.046	<0.001	0.192
	TG	LDL-C	HDL-C	Glucose	Insulin
Kendall’s Tau-b	0.087	0.003	−0.004	0.033	0.034
*p* value	0.223	0.970	0.954	0.645	0.716
	Age	Gender	BMI	MFR	ALT
Spearman’s rho	−0.09	0.063	0.186	−0.363	0.120
*p* value	0.833	0.492	0.040	<0.001	0.186
	TG	LDL-C	HDL-C	Glucose	Insulin
Spearman’s rho	0.115	0.002	−0.006	0.040	0.040
*p* value	0.210	0.985	0.945	0.661	0.742

Abbreviation: US, ultrasonography; NAFLD; nonalcoholic fatty liver disease; BMI, body mass index; MFR, skeletal muscle-to-body fat ratio; ALT, alanine aminotransferase; TG, triglyceride; LDL-C, low-density lipoprotein cholesterol; HDL-C, high-density lipoprotein cholesterol.

**Table 3:** Ordinary regression regression analysis of the association between NAFLD severity and MFR or BMI.

Severity of NAFLD (n = 122)				
	ß	SE	95% CI	*p* Value
MFR	−4.154	1.296	−6.694–−1.615	0.001
BMI	0.082	0.045	−0.006–0.171	0.069

Abbreviation: NAFLD; nonalcoholic fatty liver disease; ß, beta regression coefficient; SE, standard error; CI, confidence interval; MFR, skeletal muscle-to-body fat ratio; BMI, body mass index.

**Table 4:** TyG index and AIP of the study population according to MFR tertiles.

Parameters	Tertile 1 (Lowest, n = 41)	Tertile 2 (n = 40)	Tertile 3 (Highest, n = 41)	*p* Value
TyG index	8.75 ± 0.48	8.71 ± 0.53	8.45 ± 0.41	0.009
AIP	0.46 ± 0.22	0.45 ± 0.19	0.33 ± 0.19	0.007

Data are presented as mean ± SD. Abbreviation: SD, standard deviation; TyG, triglyceride and glucose; AIP, atherogenic index of plasma.

## Data Availability

The original data shown in the publication are openly available on GitHub at https://github.com/YOOWON-KWON/Sarcopenia_NAFLD_Rawdata.git (accessed on 16 October 2024). This dataset includes raw experimental data, and further inquiries are available from the corresponding author upon reasonable request.
